# A skeleton-based approach to analyzing oculomotor behavior when viewing animated characters

**DOI:** 10.16910/jemr.10.5.7

**Published:** 2017-12-18

**Authors:** Thibaut Le Naour, Jean-Pierre Bresciani

**Affiliations:** University of Fribourg, Switzerland

**Keywords:** Eye tracking, AOI, animated character, gaze visualizations

## Abstract

Knowing what people look at and understanding how they analyze the dynamic gestures of their peers is an exciting challenge. In this context, we propose a new approach to quantifying and visualizing the oculomotor behavior of viewers watching the movements of animated characters in dynamic sequences. Using this approach, we were able to illustrate, on a 'heat mesh', the gaze distribution of one or several viewers, i.e., the time spent on each part of the body, and to visualize viewers' timelines, which are linked to the heat mesh. Our approach notably provides an 'intuitive' overview combining the spatial and temporal characteristics of the gaze pattern, thereby constituting an efficient tool for quickly comparing the oculomotor behaviors of different viewers. The functionalities of our system are illustrated through two use case experiments with 2D and 3D animated media sources, respectively.

## Introduction

Body language plays an important role in human communication. It helps to convey and understand the emotions or intentions of others. The role of body language in human communication is actually so important that some social activities and sports (e.g., dance, gymnastics) are based on codified gestures, the production of which can be evaluated and judged by juries of experts. Along a similar line, human performance sometimes heavily relies on the ability to use current movement and / or gesture information to anticipate the future actions of others. This is the case in sports like tennis, boxing, or soccer, in which anticipating what opponents and / or partners are about to do is crucial. Therefore, a better understanding of how people scrutinize and analyze the gestures of other individuals can help improve human communication and performance.

By measuring oculomotor behavior while the viewer watches images, natural scenes or movies, eye tracking technology becomes a powerful tool for developing a better understanding of what the viewer's perceptual strategies are and how he / she gathers information. However, eye tracking can generate large quantities of data which need to be processed and analyzed in order to identify and extract the most relevant information regarding the viewer's oculomotor behavior. Processing of eye tracking data notably entails data exploration, organization and visualization, in order, for instance, to prepare datasets for statistical analysis and / or to communicate information to a non-expert public.

In this paper, we present a visualization tool designed to analyze and visualize eye tracking data from experiments with animated characters. We developed this tool to help the users better understand the perceptual strategies of viewers watching human-body gestures. The watched material can be in 2D (e.g video) or 3D format (e.g virtual reality), and our tool allows the user to process and index eye tracking data relative to movements that are semantically equivalent but do not necessarily have the same dynamics. The user can compare or aggregate data from groups of animated media sources and/or groups of viewers to spatially and temporally visualize the gaze data distribution on the different parts of the body. These operations allow the user to visualize differences (e.g., in synchronization) between viewers or groups of viewers, and compare the number and duration of the fixations. Importantly, our tool has been designed to be easily used by both experts and non-experts in eye tracking data processing.

**Figure 1.**
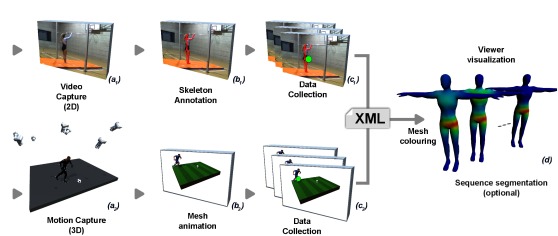
Overview of the pipeline of our system. After capturing an animated sequence by video capture (_a1_) or using a system of motion capture (_a2_), the user manually annotates (_b1_), or the captured motion is automatically annotated (_b2_). After visualization of the animated sequences by the viewer(s), our system automatically computes the mapping between each fixation and the different parts of the skeleton. Finally, our system generates one or more meshes (corresponding to a temporal decomposition given by the user) colored by heat maps of the tracked areas (d).

To illustrate the functionalities of our visualization tool, we present two use case experiments with applications in the sport domain. In the first experiment (second section), official gymnastic judges were required to evaluate gymnastics sequences. In the second experiment (third section), goalkeepers had to 'analyze' the movements of the penalty taker during the run-up to the ball in order to assess the direction of the kick. The two use case experiments are based on two different kinds of animated format: 2D videos for the first experiment (cf. Figure 1.a_1_, b_1_, c_1_) and 3D scenes in virtual reality for the second experiment (cf. Figure 1.a_2_, b_2_, c_2_). In both experiments, the visual scene consists of sequences of motion of an animated character. A 3D scene represents an animated character generated by a 3D engine (e.g., Unity 3D or Unreal engine). These sequences contain dynamic stimuli which are annotated according to the anatomical parts of the human body. As proposed by Blascheck and colleagues ( 6 ), we define these stimuli as Areas of Interest (AOIs).

## Gaze visualization on animated characters

### Related techniques

Our system of visualization relies on two concepts dedicated to the visualization of stimuli representing animated characters:

•a heat mesh (i.e., the colored mesh illustrated by the Figure 1.d) is used to provide qualitative information about the gaze distribution of one or more viewers;

•a viewer timeline is used to specifically compare the synchronization between viewers.

Blascheck and colleagues ( 6 ) proposed a classification of visualization systems according to their specific features and target applications. Based on their classification, our system belongs to the AOI-based techniques (as opposed to point-based techniques), and is designed to address spatio-temporal aspects (as opposed to systems addressing temporal-only or spatial-only aspects). Using their terminology, our system works on 2D and 3D dynamic stimuli and provides a visualization which is static, interactive, in 3D, on single or multiple users and not in context.

**AOIs.** Although our system provides a graphical output (defined by a colored mesh) similar to those of heat map ( 18 ) or vertex-based mapping ( 29 ), the colored mesh is built from data previously quantified via body area annotations, instead of directly calculating the attention map with eye tracking data. These annotations (the parts of the body) are several regions defined as dynamic AOIs ( 23 ). Areas of Interest (AOI) constitute a classical way to describe visualized stimuli. For example Rodriguez and colleagues ( 24 ) proposed to annotate a video recording of television news with different areas which are static. More similar to our study, Papenmeier and colleagues ( 22 ) proposed a tool which analyzes virtual scenes with dynamic AOI. Specifically, the authors created a 3D-model of the scene from the video and annotated the most important objects and their trajectories manually.

Along a similar line, Stellmach and colleagues ( 29 ) provided a tool dedicated to the annotation of 3D scenes and the visualization of the gaze data distribution of viewers. After annotating the different objects of the scene, they proposed the concept of models of interest (MOI) to facilitate the investigation of objects in 3D scenes by mapping the data against time.

In an industrial context, several companies (which sell eye trackers) proposed tools which allow to work with dynamic stimuli (i.e Tobii, SMI).

While most of these tools work well with simple animations, they cannot be used to analyze complex skeleton-based animations, such as those involving human movement. Our tool has been specifically developed to fill this gap.

**Timeline AOI visualization.** Timeline visualization is used to show the temporal aspects of AOI-based data. Classically, time is represented on one axis while AOIs or viewers are represented on a second axis with separate timelines. Similar to our method, Wu ( 35 ) used timelines colored with references to the scene objects for each viewer. Kurzhals ( 14 ) proposed a framework 'unifying AOI and viewers timelines’. However, understanding these unified representations becomes increasingly difficult when the number of viewers and AOIs increases.

The main benefit of our approach lies in the fact that all AOIs (i.e parts of the body) are summarized in a unified model, relying on an articulated mesh. This representation allows us to graphically decouple the timelines of viewers for each AOI (cf. Figure 4). As we will see, our method also allows to easily compare differences of synchronization between viewers.

**Gaze behavior on characters.** Although a large number of studies rely on the analysis of gaze behavior with stimuli represented by animated characters, applications allowing the exploration and visualization of the data collected are often overlooked. Bente and colleagues ( 3 ) were among the first authors to use eye tracking to quantify the time spent on animated characters. In an experiment investigating nonverbal behavior, they compared the participants' impression regarding a sequence of dyadic interactions in two different contexts: video recording vs computer animation. To do that, provided that their characters remained in a very restricted area, they recorded the time spent on 6 fixed areas: upper area (head and facial activity), middle area (body, arm, and hand movements) and lower area (leg and foot movement) of the two characters. More recently, still investigating nonverbal behavior, Roth and colleagues ( 25 ) used annotated rectangle AOIs on videos to quantify the time spent on head and body regions.

### Our visualization system

In the following section, we first present the features of our system and then we detail how it was designed. The subsequent sections will present two use cases based on 2D and 3D media sources, respectively.

The main characteristics of our visualization system are: First, it is dedicated to the visualization, aggregation and comparison of eye tracking gaze data for one or several viewers who can be split into different groups. Second, unlike most software, it is independent of both the hardware used to collect the eye movement data and of the application used to generate the visual stimuli. Third, it can be easily used by both experts and non-experts in oculomotor research.

**Input data.** With our system, eye tracking data can be collected both with video or 3D media sources. The user has to provide two types of information as input to our visualization system: the experimental material which is common to all viewers, and the recorded eye movement data of each individual viewer. This information has to be provided in XML format (see the example given in the supplementary material).

**Experimental material.** The experimental material consists of the viewed material and a list of media sources descriptions (i.e., metadata) used for the experiment. In our case, the viewed media sources represent movements of real characters in videos and virtual characters in 3D. All viewed media sources need to be semantically equivalent but they do not need to have the same kinematic features. The information describing a media source can be divided into three categories:

•**Basic information.** Name and duration of the media source; Note that the name is important since the user will be able to compare different media source by class, by associating one or various parts of the name to different classes.

•**Intervals (optional).** The user has the possibility to define specific intervals. In most cases, the movements that we want to analyze can be segmented into temporal subsequences. Specifically, a movement can be semantically or technically viewed as a concatenation of different phases, each of which can be analyzed separately. For example, whether for interpersonal communication or in sport situations, each 'phase' can be analyzed separately. The resulting subsequences are defined manually by the user according to the more general context of the analyzed gesture. The result of this partitioning is illustrated in Figure 1.d.

•**Temporal mapping between media sources (optional).** The user can add a temporal mapping between media sources. As mentioned before, one of the features of our system is the ability to compare the gaze distribution of various viewers on different semantically equivalent gestures. Segmentation is a first step in this direction. However, for any given piece of semantic information, the gestures present different kinematic features (in particular regarding speed and acceleration, or the beginning and the end of movements). To overcome this problem, our tool gives the user the possibility to create a temporal correspondence between any chosen reference sequence of movement and any other selected sequences. This temporal correspondence is performed by the dynamic time warping algorithm. This process is explained in detail in the third section.

**Viewers' experimental information.** Viewers' information consists of a list of captures for each viewer. Each capture is defined by three types of information:

•**Basic information.** The name and the index of the capture order during the experiment. Note that the name is important since the user will be able to compare different media sources by groups, by associating one or various parts of the name to different groups;

•**Eye tracking samples.** This is a list of eyetracking samples. Each sample is defined by the coordinates of the eyes onto the screen and its corresponding time;

•**Sample joint mapping.** For each sample, an AOI is defined either by the name of the body part of the viewed character, or by an empty string. The list contains n values. Regarding the naming of body parts (i.e. joints of the skeleton), our system accepts the classical nomenclatures used by the major software products such as motion builder, mixamo, unity or kinect. In the next sections, we will explain how we calculate this sample body part mapping in 2D and 3D.

**Visualization features.** In this section, we explain how we combine the spatial and temporal information related to one or several viewers (in the same representation).

**Fixation calculation.** From the list of samples related to each capture, only fixations are used to compute visualization information. As described by ( 26 ), we consider a fixation as 'a pause over informative regions of interest' which lasts >100ms and has a spatial dispersion of gaze points with a threshold set at 1°. To extract this information from the list of samples, we use the Dispersion-Threshold Identification (I-DT) algorithm ( 28, 30 ). Then, from the mapping between eye coordinates and intersected body parts, we compute a new fixation-joint mapping which corresponds to the fixations which intersect an AOI area on the character. From this new mapping, we obtain a fixation time T(j) for each joint. Note that if we calculate the sum T = sum_{j in K} T(j), T is not equal to the global fixation duration of S since we exclude eye tracking coordinates that are too far away from the skeleton as well as data recorded during saccadic eye movements.

**Spatial information.** One of the main techniques used to visualize gaze patterns is known as heat maps. Introduced by Mackworth and Mackworth in 1958 ( 18 ), this simple and intuitive technique provides qualitative information about the gaze behavior of the viewers. In 3D, some studies ( 20, 29 ) used attention maps on 3D objects (object-based or surface-based). The graphical results provided by our method look like those provided by a surface-based method, but the difference between the classical studies and our method lies in the fact that we build the heat map from data previously quantified via body area annotations, instead of directly calculating the attention map with eye tracking data.

In our system, the color is defined based on the concept of skinning, commonly used in computer graphics. Skinning ( 17 ) is a process which associates each vertex of a mesh with one or several joints of a skeleton. A weight is then attributed to each joint bound with a vertex. Usually, this technique allows to naturally animate and deform a mesh controlled by a skeleton. For example, the vertices located around a joint and influenced by two bones are impacted by the weighted transformations of two joints. With our system, the mesh is colored according to these weights and the joints attached to each vertex. This step creates a shading between the different parts of the body. Thus the scalar color of a vertex v_c_ is calculated as:

**(1):**
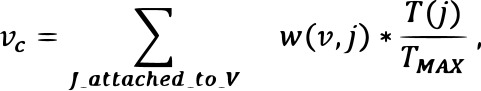


where w(v, j) is the weight which associates the vertex v and the joint j. To have a good overview of the distribution of the data, we propose three metrics to calculate T_MAX_. First, T_MAX_ represents the total duration of the interval concerned (i.e fixations in and outside of the mesh, and saccadic movements). Second, T_MAX_ represents the sum of T(j) only on the character during a given interval. Third, T_MAX_ represents the maximum value calculated on every part of the body (during a given interval).

**Figure 2.**
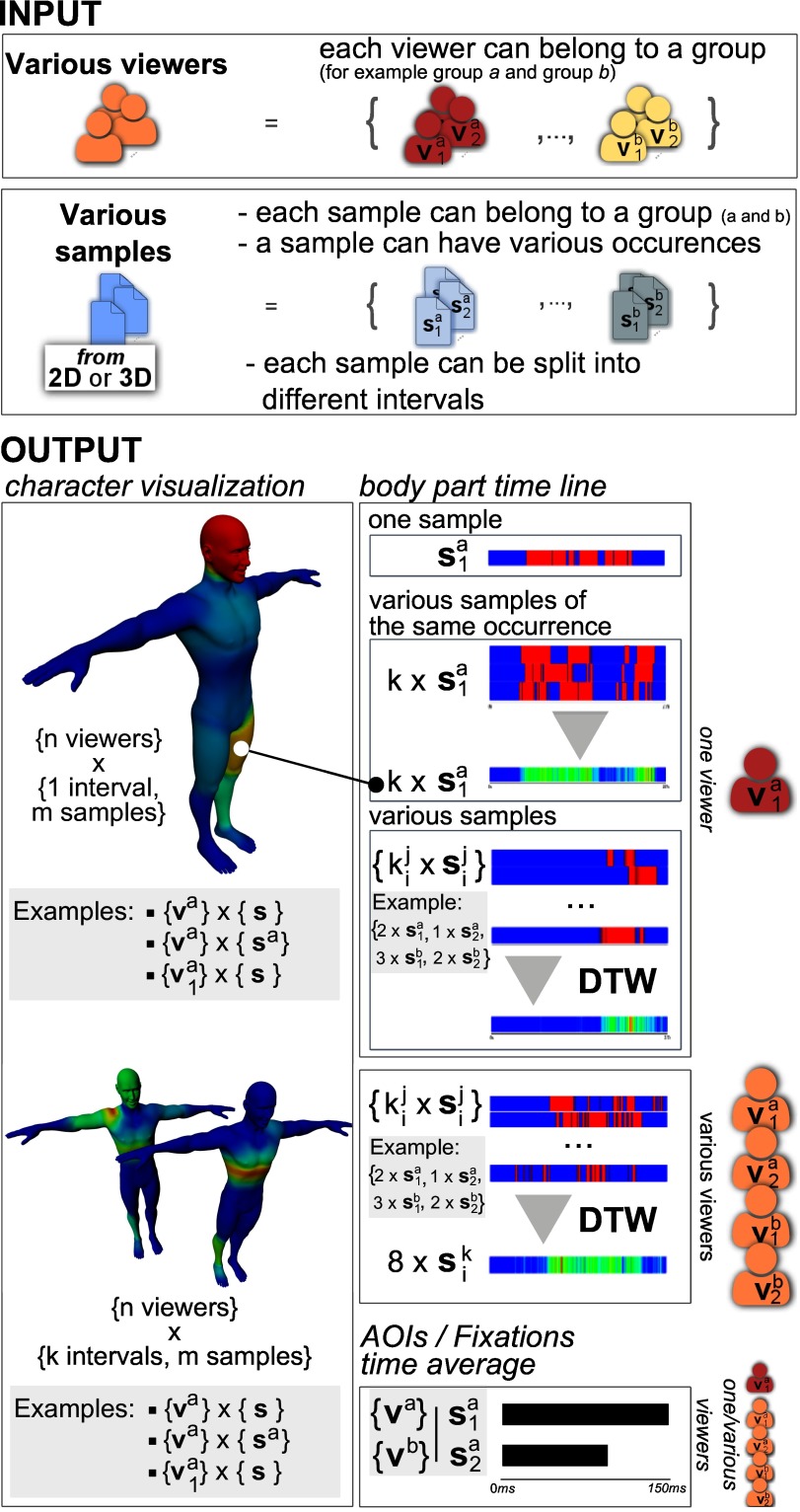
Overview of the different features of our system.

Figures 1.d and Figure 2 (Output/left column) show examples of the mesh coloring for various viewers with a dynamic motion picture, partitioned into sub-sequences (Figure 1.d).

In the case of multiple viewers, the mesh color is obtained by averaging the fixation durations T_viewer_(j) (for each viewer ) and applying Equation 1. The result is illustrated in Figure 2 (left column at the top).

Our skinning definition contains 51 joints divided into: 5 joints for the body trunk, 2 joints for the neck and the head, 4 joints for each leg, 3 joints for each arm, 15 joints for the fingers of each hand.

**Timeline visualization.** In line with recent work on viewer timeline visualization ( 14, 35 ), our system represents spatial and temporal features of viewers' gaze patterns in a single figure. The originality of our contribution lies in the decoupling of areas into separated blocks of timelines. Thus, for each block, we present separate timelines stacked horizontally, as well as fixation percentages by viewer and watched body part.

As illustrated in Figure 2, we propose various options to visualize the temporal information. First, as for spatial information, we only use the fixations to calculate the temporal visualization. Second, we distinguish two modes of timeline visualization: single or aggregated. In the **single mode**, each timeline represents one motion picture. The distribution of the watched body part is represented by two colors: red when the region is watched and blue when it is not watched. In the **aggregated mode**, a timeline represents the gaze distribution on several motion pictures. Here we use a 'heat' line to represent the information. If the motion pictures to aggregate are the same (i.e we want to aggregate various occurrences of the same motion), we just average the set of occurrences for each time step of the captured gaze pattern. If the motion pictures / sequences are not the same (but semantically equal), we use the correspondence mapping previously introduced to aggregate them. Specifically, for each viewer, the color of each value on the timeline is normalized by the maximum value calculated among all parts visualized (this, for a given interval).

As explained before, the user can choose to visualize several media sources or/and several viewers, and each media source can be represented by several intervals. So, considering the two modes of visualization previously introduced, several visualization possibilities are available. For one viewer and a given interval, it is possible to display, for each body part, either all captures, or the aggregation of all occurrences of the same media source, or the aggregation of all media sources. Additionally, the user can filter or compare various media sources. For several viewers and a given interval, the options are the same but the user can also compare or filter the viewers.

**AOIs / Fixations visualization.** The last proposed feature concerns the visualization of the "time spent" on body parts (Figure 2 (Output/bottom of the right column)). Considering a given interval, for each AOI, it is possible to display the number and the average duration of fixations, as well as the number and duration of the sum of adjacent fixations on this AOI. The selecting options for AOIs/Fixations visualization are the same as for timeline visualizations.

## 2D use case: evaluation of gymnastics judges

Eye tracking plays a central role in developing a better understanding of the relationship between gaze behavior and decision making in sports. In this context, the aim of this experiment was twofold: First, investigate how gymnastics judges analyze a gymnastic gesture, and second, determine if there are differences in the gaze pattern of judges of different levels of expertise.

While eye tracking is often used in sport applications to analyze perceptual-cognitive skills, sports official are often overlooked. Hancock and colleagues ( 10 ) recently performed an experiment assessing gaze behavior, decision accuracy, and decision sensitivity of ice hockey officials. In gymnastics, Bard & al.( 1 ) conducted a study analyzing the gaze pattern of gymnastics judges. They specifically measured the number and location of ocular fixations. They found that experts had fewer fixations of longer duration. This has been confirmed by several studies in gymnastics ( 31 ), in handspring vaults ( 21 ), and in rhythmic gymnastics ( 13 ).

In our first use case experiment, official gymnastics judges had to evaluate and mark the performance of gymnasts on the horizontal bar. Video footages of gymnasts' performances were shown to the judges and we recorded their eye movements. This kind of animation material constituted an excellent benchmark for our system because: (i) each sequence can be segmented into several phases; (ii) the movement is rich in information (the character uses all parts of his/her body and rotates around him/herself); (iii) finding out whether oculomotor behavior differs between participants and trying to link these behaviors with the attributed marks has a functional relevance.

### Material preparation

To work with video sequences, we created an application to annotate them manually by adding a skeleton which follows the video character, i.e the AOIs are defined by the skeleton (Figure 1.b shows an example of the 2D skeleton mapped on the related video material). The skeleton creation and annotation were performed by one user, a member of the research team. In this experiment, the skeleton contained 26 joints and 30 segments which were set up with a width and a depth level. The depth level notably determines which segments are displayed on the front plane in case of 'overlap', thereby managing masking between objects. The number of joints corresponding to a low-resolution skeleton (less than the 50 joints possible introduced in the previous section) was arbitrarily defined to simplify the task for the user. This aspect constitutes a direct limitation of the use of video format, i.e., it is impossible to have a high resolution of the subjacent skeleton. The skeleton annotation required approximately 1 hour for every 30-second sequence of video. On average, the user annotated a skeleton every four frames, the remaining animation being automatically computed by interpolation between the annotated frames.

Figure 1 (upper part) provides an overview of the experiment.

**Figure 3.**
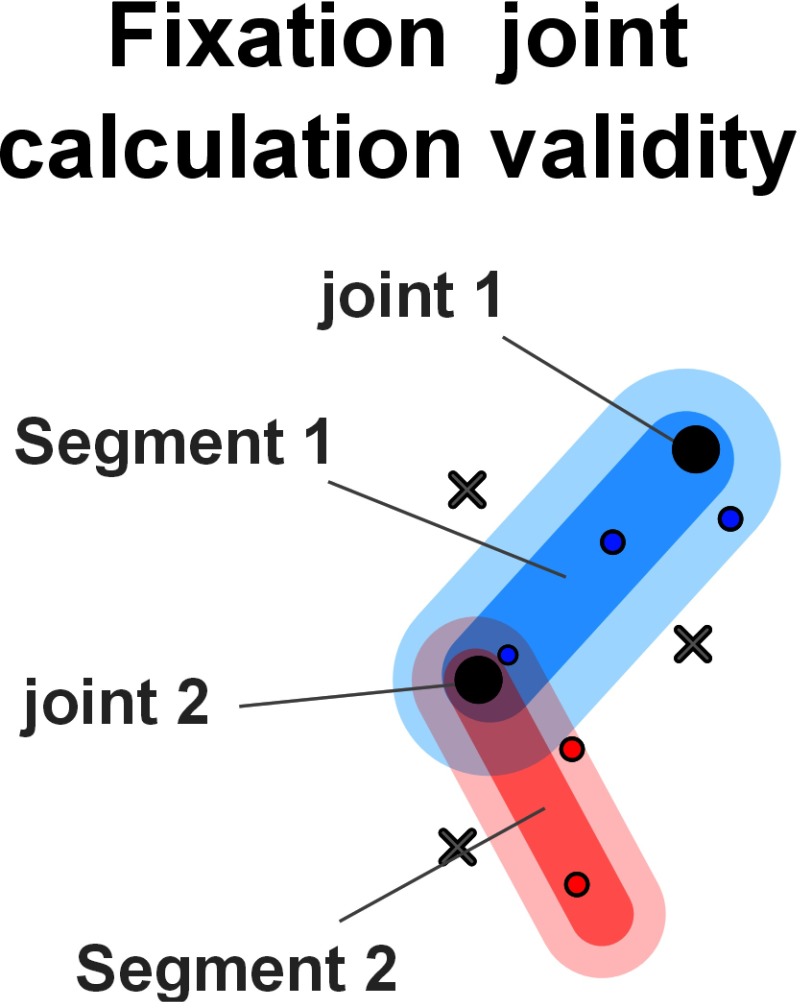
Only the samples on and near the segments are taken into account (represented by blue and red dots, respectively). Here for instance, the crosses are too far and will therefore not be taken into account.

**Eye gaze-sample joint mapping.** As mentioned before, to calculate gaze data distribution, the visualized body part must be provided for each sample. The sample joint metric that we used is similar to existing metrics used to calculate mean gaze duration and proportion of time spent on each dAOI ( 12 ), here represented by the areas linked to the joints of the skeletons (see Figure 3). For each frame of the sequence, we measured the eye tracking coordinates on the screen c_k_ = (c_k_^x^, c_k_^y^) where k represents the current frame. The associated joint is determined by a function f which calculates the shortest distance between the bones of the joints of the skeleton as well as the coordinates c_k_. As illustrated in Figure 3, f excludes the coordinates which are too far away from the skeleton. This distance of exclusion is directly given by the user accordingly to the accuracy of the eye-tracker used. In our case, the accuracy of the system allowed us to use a distance of 0.25° to 0.5°. If the coordinates are valid, f calculates the best-fitting bone, depending on the shortest distance between skeleton bones and the coordinates c_k_ and taking into account the depth hierarchy between joints.

### Experiment

We used the SR Research Eyelink 1000 Plus eye tracker to record eye movement data, sampling at 1000 Hz. The angular distance between the two adjacent 'closest' joints was 3.05°and the distance between the two adjacent 'most distant' joints was 8.1°.

**Participants.** 18 female gymnastics judges participated in the experiment. Nine judges had a Swiss level B.1 (M_age_ = 24.5 years, SD = 2.3 years) and the other nine a Swiss level B.2 (M_(19)_ = 32.8 years, SD = 5.6 years). The B.2 judges had more experience and expertise than the B.1 judges (they need to judge 6 competitions before starting the B.2 training which lasts one year).

**Procedure and Design.** Each judge was presented with 9 videos of gymnasts performing a movement at the horizontal bar. The videos were filmed with 3 gymnasts of different levels (C5, C6 and C7 of the Swiss Gymnastic Federation) who performed 3 occurrences of the same gesture. The point of view of the videos corresponded to the placement of judges during a competition. Each video lasted about 30s (~900 samples with a frequency of 30 frames/second and ~200 annotated skeletons). During the experiment, viewers were placed in an ecological situation (i.e the distance between the viewer and the video character corresponded to what occurs during a competition). Two repetitions of each video were presented, for a total of 18 videos per judge. For each judge, the order of presentation of the videos was counterbalanced using a Latin square.

**Figure 4.**
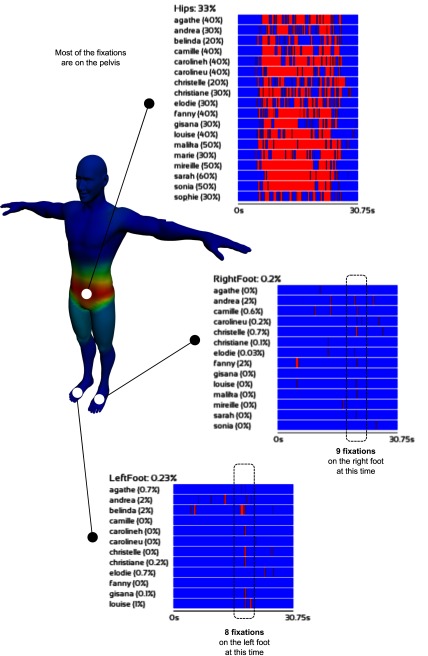
Illustration of viewers' gaze patterns. The colored mesh averages the data recorded with 18 viewers. Each block represents a body part and each line corresponds to a viewer (the red color shows the fixation times). The blue color means that the area is not watched at this time. The block percentage corresponds to the average time spent on the related body part by assuming that we considered only fixations on the body. The timeline percentage corresponds to the average time spent on the related body part. Specifically, these two percentage values have been calculated on all sequences.

**Results.** Figure 4 shows the results of randomly selected sub-sequences for two different kinds of body part: a body part that was often visualized (the hips) and body parts that were seldom visualized (right and left foot). First, we can note that the heat mesh illustrates with clarity the areas of interest (gazed at) in the selected subsequences for one or more viewers.

Concerning global fixation, as expected for gymnastics experts, our results show that the body part that was the most scrutinized by the judges was the hips area, which is a central 'anchor' to evaluate performance in gymnastics.

Concerning the timeline blocks visualization, Figure 4 illustrates clearly the differences in synchronization between viewers for a given sample. Specifically, a viewer's timeline represents a sub-sequence of the movement, and is linked to a body segment / area. The red color indicates the time slots during which a given segment / area is gazed at. It is particularly interesting to notice that for definite areas, the majority of judges are 'synchronized' when fixing their gaze on peripheral areas. After verification, these areas correspond to movements that were not executed correctly, i.e., movements that can be considered as ‘artifacts’. Therefore, our system allowed us to easily detect mistakes or incorrectly executed movements, as well as elements and details that were missed by a judge. Gaze patterns related to the 'left foot' and 'right foot' evidence shared oculomotor 'strategies' between viewers, as several of them show synchronized fixations. The percentages associated to each timeline confirm the observed tendencies. A comparison of the two groups of judges with a general overview of gaze and fixation distribution is presented in Appendix Figure 8.

For illustration purposes, we assessed whether the level of expertise of the judges could affect the synchronization between viewers for the body parts that were infrequently scanned, namely less than 1% of the total scanning time. We considered that these fixations likely corresponded to detected errors. We expected the judges to look at these parts simultaneously because they detected the errors when they occurred. We also expected more expert judges to show more synchronization in this errordetection process. To assess that, we first computed the total overlap duration for these body parts, i.e., the total duration during which at least a third of the judges were simultaneously watching these body parts. We then compared the overlap duration between the two groups of expertise.

The more experienced judges (B.2 level) had an average overlap of M_overlap_^B2^ = 83 ms, SD_overlap_^B2^ = 78 ms, whereas that of less experienced judges (B.1 level) was of M_overlap_^B1^= 76 ms, , SD_overlap_^B1^ = 49 ms. However, a Welch two sample t-test (data normally distributed and homogeneous variance between groups) indicated that this difference between the groups was non-significant (t(14)=0.222, p=0.827). Once again, this comparison was performed for illustration purposes, and many more tests could be run on the data depending on the specific question at hand.

## 3D use case: Prediction of the direction of a penalty kick

In the second use case experiment, goalkeepers had to scrutinize the run-up of the penalty taker to try to determine ahead of the kick in which direction the ball would be kicked. This experiment was conducted in virtual reality.

Sport applications based on virtual reality technology are increasing almost exponentially. In particular, VR is very useful to analyze players / athletes' behavior or to improve sensorimotor learning. For example, Huang ( 11 ) proposed an application to improve American football performance based on gameplays created by coaches. A survey dedicated to the use of virtual reality to analyze sport performance is given by Bideau and colleagues ( 5 ). In our use case experiment, the motion picture of the penalty taker was in 3D format, namely a 3D animation. As explained by Bideau and colleagues, this format presents several advantages over video playback. In particular, it allows for easy editing of the scenes and adding embedded 2D and 3D information. It also allows to flexibly edit the movement to modify the dynamic, the orientation of the joints, the display of certain parts of the body, or the juxtaposition of several movements. Concerning the use of both virtual reality and eye tracking for the analysis of animated characters, Bente et al. ( 3 ) were among the firsts to compare the effects of videotaped nonverbal interactions vs computer animations of the same behavior on perception. They found that the two media sources give rise to similar results. In their study, the animation of the virtual characters was based on interpolations between wire-frame models which did not move. Since Bente, several studies combined virtual reality and eye-tracking for research on social interactions. For example Lahiri & al. ( 16 ) used virtual reality to provide dynamic feedback during social interactions. They wanted to better understand the gaze patterns of adolescents with autism spectrum disorders. To visualize data, they used the classical scanpath and AOI techniques as introduced by Rogriguez ( 24 ). In another study, Bente and colleagues ( 2 ) investigated the length of gaze fixations during virtual face-to-face interactions. More recently, Roth et al. ( 25 ) analyzed the impact of body motion and emotional expressions in faces on emotion recognition. They compared the visual attention patterns between the face and the body area and found that humans predominantly judge emotions based on the facial expression. In another context, Wilms and colleagues ( 32 ) used the gaze behavior of viewers interacting with a virtual character to modulate the facial expressions of the virtual character (i.e., the facial expressions of the virtual character depended on the gaze behavior of the viewer).

As mentioned in the previous section, eye tracking technology is often used to analyze gaze behavior to better understand decision making with athletes, coaches and officials. Concerning the striker-defender opposition, several studies (e.g., in tennis ( 9 ), or in soccer ( 19, 31 )) have shown that expert players who make faster decisions and have more correct responses make more fixations. Hancock and Ste-marie ( 10 ) suggested that fixation duration depends on the type of sport and the experimental conditions. Specifically, shorter durations are usually observed for interception (e.g., racket sports) and strategic sports (e.g., team sports) whereas longer durations take place in closed-skills sports. This has been confirmed by Woolley et al. ( 34 ) who investigated goalkeeper anticipation during a penalty kick and observed fewer fixations of longer duration on fewer locations. However, to our knowledge, no study has yet explored the differences and common synchronizations between viewers or groups of viewers.

Anticipating the direction of the ball during a football penalty kick is a topic which has often been investigated. For instance, Savelsbergh and colleagues ( 27 ) have shown that goalkeepers were more successful in anticipating the direction of the kick to come when fixating on the stance leg (i.e., non-kicking leg) prior to foot-to-ball contact. In contrast, Woolley et al. ( 34 ) suggested that when trying to predict the direction of the kick, goalkeepers could use a global perceptual approach by extracting information cues from various body segments of the body (e.g., kicking leg, stance leg, hips) rather than focusing on one particular body area. In our use case experiment, we assessed whether there are differences between novice and expert goalkeepers regarding the visual scanning strategy used to anticipate the direction of the kick, and how those affect the estimation performance.

### Experiment

**Figure 5.**
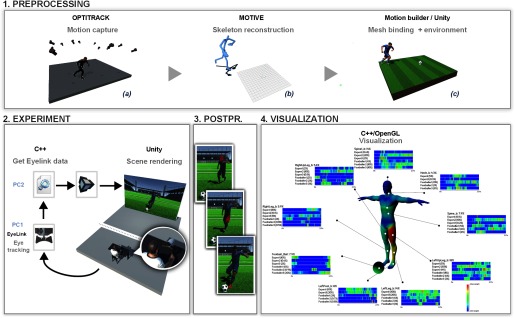
Overview of the steps and design of the second use case experiment assessing goalkeepers' performance and strategies in anticipating the direction of a penalty kick.

The steps usually involved in the 'production' of a motion picture in 3D format and its use in a virtual reality setup are illustrated in Figure 5. As shown, several preprocessing steps are required. The preparation of the 3D scene is decomposed into three steps: the capture of the actors (1.(a)), the creation of an animated skeleton (1.(b)) and the creation and binding of a mesh to this skeleton: 1.(d).

To capture the gestures of the actors, we used the Optitrack system with 12 infrared cameras rated at 240 Hz. The actors were dressed in a suit equipped with 49 markers. The animation of the skeleton (composed of 51 joints) was automatically computed by the Motive software. To create a mesh and bind it to the animated skeleton, we used the Mixamo and MotionBuilder softwares, respectively. Mesh creation takes about fifteen minutes, and five additional minutes are needed for every gesture. Finally the virtual scene was create and rendered with Unity3D.

Twenty penalty kick gestures were motion-captured with five football players having on average fifteen years of experience in football. Each player performed four gestures, namely two kicks to the right side and two kicks to the left side. Each gesture included the full sequence of a penalty kick: laying of the ball, preparation, run-up and ball kick. For each trial, the whole sequence was presented to the viewer, but only the run-up and the kick phase interested us for the analysis. Because the original sequences had different durations, the Dynamic Time Warping algorithm (DTW) was used to create a correspondence between the sequences. Following this operation, all sequences 'fed' to our visualization system are mapped to the duration of the reference sequence.

**Figure 6:**
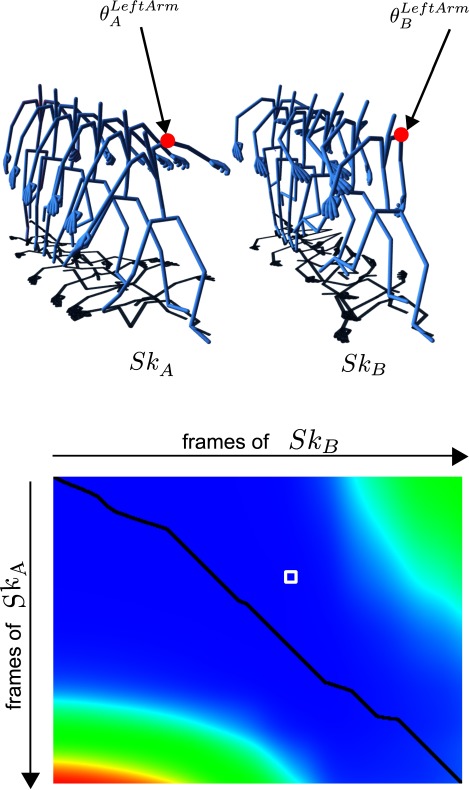
Skeleton animation for two different gestures (top) and time warping matrix for these two animations (bottom). The blue areas correspond to the zone for which the distance between the posture of Sk_A_ and that of Sk_B_ is the lowest. The black curve represents the warping path.

**DTW.** When all movements do not have the same duration, and when the gestures have different dynamics and characteristics (e.g., different count of footsteps during the run-up in our experiment), it is impossible to establish a linear mapping between the motion pictures. To temporally map the different motion pictures, we used the dynamic time warping algorithm introduced by Berndt and Clifford ( 4 ) on time series and applied in computer animation by Bruderlin and Williams ( 7 ). This algorithm calculates an optimal match between two given gestures. First, the algorithm calculates the matrix of distances (see Figure 6) between the postures of two given gestures. Next, it calculates the optimal path corresponding to the best temporal warping to align the two sequences.

In this study, we applied the algorithm to the analysis of 3D animations by using the distance function

**(2):**
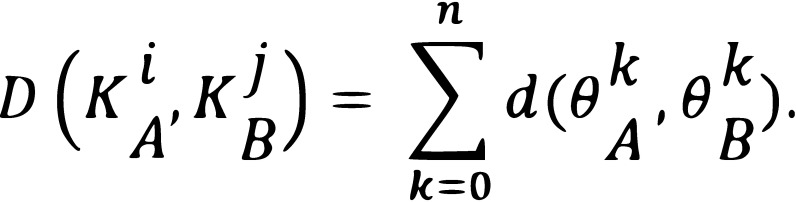


In this equation, K_A_ and K_B_ are the two skeletons which are compared, i and j are the indices of the postures in the sequences A and B, n the number of joints in the skeleton and the angle (represented by a quaternion) of the current joint. d = ‖Log(θ_A_^-1^θ_B_‖ represents the geodesic distance ( 8 ) between two quaternions θ_A_ and θ_B_. In Figure 6, D is represented by the black square. This procedure allows to compare various gestures. The next section explains how we calculated the time spent on each part of the body.

**Eye gaze-sample joint mapping.** After collecting the gaze data, we used the OpenGL library ( 33 ), provided by Unity3D, to map the eye coordinates and the objects displayed in the scene. Specifically, we used a ray cast algorithm to project a 3D ray from the gaze screen coordinate through the camera into the scene, and then check if that ray intersected any body parts. For each gaze sample collected at a specific time, we played the scene and used this algorithm to map the sample to a body part.

**Participants.** Football players having between ten and twenty years (Mexp=15.4 years) of football experience participated in the experiment.

Five of these players were expert goalkeepers (M(19) = 23.5 years, SD = 1.7 years) whereas the other five were expert field players (M(19) = 23.9 years, SD = 3 years) without any goalkeeping experience.

**Procedure and Design.** The participants were presented with two repetitions of the twenty different penalty kick sequences, for a total of forty sequences per participant. The order of presentation of the sequences was fully randomized for each participant. Because the orientation of the head before the run-up sometimes provided important information regarding the future direction of the kick, the animation of the head was altered so that it did not provide any information regarding the kick. This modification was implemented because we were interested in the gaze pattern of the participants during the runup phase. For each trial, participants had to estimate whether the kicker would strike to the right or to the left of the goal, and give their response as fast as possible (i.e., as soon as they made their decision) by pressing a response key.

### Results

Regarding anticipation performance, we compared the results of the expert goalkeepers (GK) with that of the field players (FP) both regarding the percentage of anticipation error and the time of the response. On average, goalkeepers had an error rate of M_error_^GK^ = 38%, SD_error_^GK^ = 9.3, whereas the field players had an error rate of M_error_^FP^ = 38.5%, SD_error_^FP^ = 8.9. A Welch two sample t-test (data normally distributed and homogeneous variance between groups) indicated that this difference between the two levels of expertise was non-significant (t(8)=0.086, p=0.932). Regarding the time of response, goalkeepers responded on average in M_time_^GK^ = 13 ms, SD_time_^GK^ = 172 ms, before ball contact, whereas the field players responded on average M_time_^FP^ = 14 ms, SD_time_^FP^ = 126 ms before ball contact. Once again, a Welch two sample t-test (data normally distributed and homogeneous variance between groups) indicated no significant difference between the two levels of expertise (t(8)=-0.004, p=0.996).

**Figure 7.**
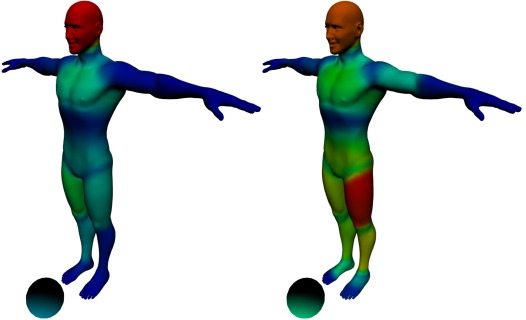
Difference of visual fixation between field players (left) and expert goalkeepers (right). The color scale represents the total duration of fixations on the different body parts

Concerning gaze behaviors, Figure 7 displays the distribution of gaze fixations recorded during the run-up of the kicker. Field players are characterized by a more diffuse visual scanning on the body and particularly on the head as compared to expert goalkeepers who focused their gaze primarily on the supporting leg. These results are in line with previous work on this topic though the setup and methodology were different ( 27 ). However, after analyzing the graphical results of the top three expert subjects, unlike the study on gymnastics, a gaze pattern is not obvious to establish.

In line with the results reported in previous studies, and for illustration purposes, we compared the gaze behavior of expert goalkeepers vs field players. In particular, we focused on the total duration of fixation as well as the total number of fixations on the lower part of the supporting leg. For this analysis, we excluded the participants that did not look at this body part at all. In total, goalkeepers spent M_duration_^GK^ = 3763 ms, SD_duration_^GK^ = 2670 ms fixating the lower supporting leg, whereas the field players only spent M_duration_^FP^ = 211 ms, SD_duration_^FP^ = 97 ms. However, a Welch two sample t-test (data normally distributed and homogeneous variance between groups) indicated that this difference failed to reach significance (t(3.0105)=2.6581, p=0.076).

Regarding the total number of fixations, on average, the goalkeepers performed much more fixations M_number_^GK^ = 11, SD_number_^GK^ = 7.9 than the field players M_number_^FP^ = 1.33, SD_number_^FP^ = 0.58. But, as for fixation duration, this difference failed to reach significance, as indicated by a Wilcoxon rank sum test (data normally distributed but heterogeneous variance between groups, W=11.5, p=0.0718).

Though non-significant, our results regarding fixation duration are consistent with those reported in previous studies. Indeed, the experts had longer fixation durations on the supporting leg. However, we also found that experts had a greater number of fixations, which is at odds with previously reported results showing the opposite pattern (appendix Figure 9). This discrepancy is intriguing and should be investigated more specifically in future research. In particular, it might be interesting to compare more systematically 3D stimuli and 2D video media sources to test to which extent they affect the gaze pattern of viewers.

## Conclusion and discussion

In this paper, we presented an interactive 3D tool, based on AOI data, that allows for spatio-temporal investigation of large data sets of recorded eye movements. In particular, this tool allows the user to manage and analyze the gaze pattern of several viewers on several animated sequences. This tool is independent of the hardware used to record the eye movements and of the application used to generate the visual scene / experimental stimuli, making it more flexible for general usage.

Specifically, we presented a new approach to visualize the oculomotor behavior of viewers watching the movements of animated characters in dynamic sequences. This approach allows to illustrate the gaze distribution of one or several viewers, i.e., the time spent on each part of the body on a 'heat mesh'. This is in line with previous work on heat maps ( 6 ) or surface-based attention maps ( 29 ). Associated to this approach, we also proposed a new way to visualize viewer timelines using blocks of timeline linked to the heat mesh. As with classical systems, our system allows to visualize the 'heat' information ( 20, 29 ) and to have a good overview of the observed AOIs ( 15, 20 ). To our knowledge, the system presented here is the first which proposes to visualize the spatio-temporal features of the gaze patterns of several viewers having watched animated characters within a unified figure. As with the interactive applications proposed by Stellmach ( 29 ) and Maurus ( 20 ), our tool allows the user to visualize the spatial distribution on 3D objects. However, we introduce a graphical link between the timelines and spatial information ( 15, 20, 29 ), and provide a clear visualization of the synchronization and overlap between viewers. Our tool also allows the user to directly export the observed features for statistical analysis. These differences are summarized in Table 1.

**Table 1.** Comparison of the features of our system with those of systems used in previous related works.

	Display feature				stimuli			
	spatial distribution	timelines	Synchro overlapping	scanpath	type	AOI	Comparing Several users	Comparing Several media
Maurus	Yes	No	No	No	3D	no	No	No
Stellmach (Tobii 1750)	Yes	Yes	No	Yes	3D	Automatic (vertex based)	No	No
Kurzhal (Tobii T60 XL)	No	Yes	Yes	Yes	2D	rectangles	Yes	No
Roth (SMI RED-500)	Yes(2D)	No	No	No	3D	rectangles	No	No
Maurus	Yes	Yes	Yes	No	2D/3D	2D: rectangles 3D:automatic (vertex based)	Yes	Yes

While our model is not specifically dedicated to the analysis of the AOI order (scanpath), the two use case experiments highlight its advantages. Regarding the first use case experiment, whereas classical studies in this domain focused on the number and the duration of fixations, our system allowed us: (1) to observe if a pattern of gaze exists not by using scanpath but by comparing the synchronization and the overlap between viewers' timelines; and also (2) to easily compare the synchronization between viewers. In the second use case experiment, our tool allowed us to clearly see the difference between expert goalkeepers and field players regarding the time spent on the different body parts. More interestingly, it allowed us to identify that the gaze behavior on certain body parts is not consistent with the literature.

These two use case experiments demonstrate that our system is an efficient tool to quickly compare the oculomotor behavior of different viewers, and notably to identify the synchronization (or the lack of it) between viewers for each dynamic area of interest. In this respect, we believe that our system is ideal for users who want to quickly and easily compare the gaze pattern of different viewers or groups of viewers.

Therefore, our system could serve as an excellent support for experiments dedicated to nonverbal behavior analysis, as for example in the study of Roth and colleagues ( 25 ) who analyzed how viewers look at avatars displaying affective behavior. Our system would notably have allowed these authors to compare the time spent on the body and the head, respectively.

Another straightforward application of our system is in research on cognition and learning in sport. Specifically, our system could be used to provide feedback to novices to help them better understand the difference between their strategy and that of experts, and to adapt their strategy accordingly to improve their performance. In other words, we believe that our system is particularly well-suited for improving performance in applications for which visual scanning strategies play a key role. These last points are all the more true as we believe our system is usable by a non-expert public.

In future work, we plan to improve the number of AOIs by including the different areas of the face. Moreover, we project to include an automatic analysis of the data allowing to compare the differences which are significant (using Test-T or ANOVA) between groups of viewers and/or groups of media sources. Concerning potential applications, after using our system in the sport domain (e.g., for judging or evaluating the interactions between players), we plan to test it in conversational situations, as for example with sign language.

### Ethics and Conflict of Interest

The author(s) declare(s) that the contents of the article are in agreement with the ethics described in http://biblio.unibe.ch/portale/elibrary/BOP/jemr/ethics.html and that there is no conflict of interest regarding the publication of this paper.

## Figures and Tables

**Figure 8.**
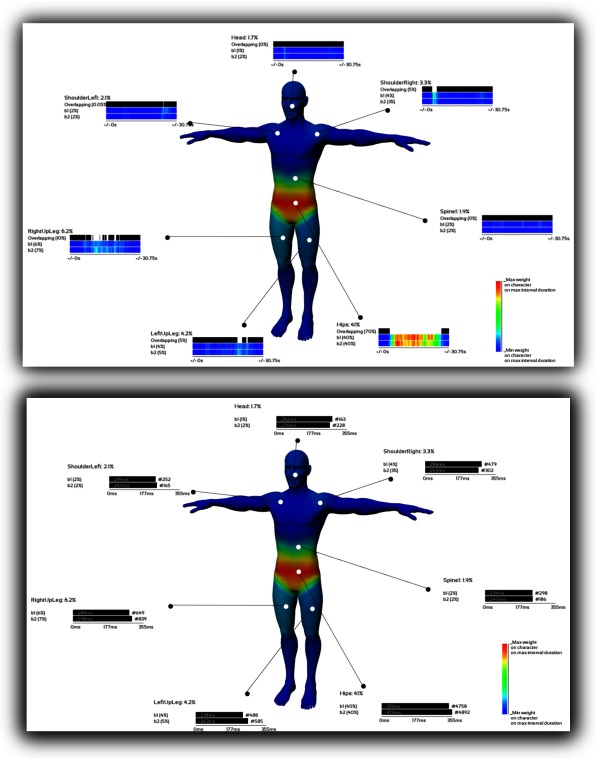
**Gymnastic judgment.** Top: Illustration of gaze distribution average for the two groups of judges (level B1 and B2). Bottom: Illustration of viewers' fixations for each group of judges.

**Figure 9.**
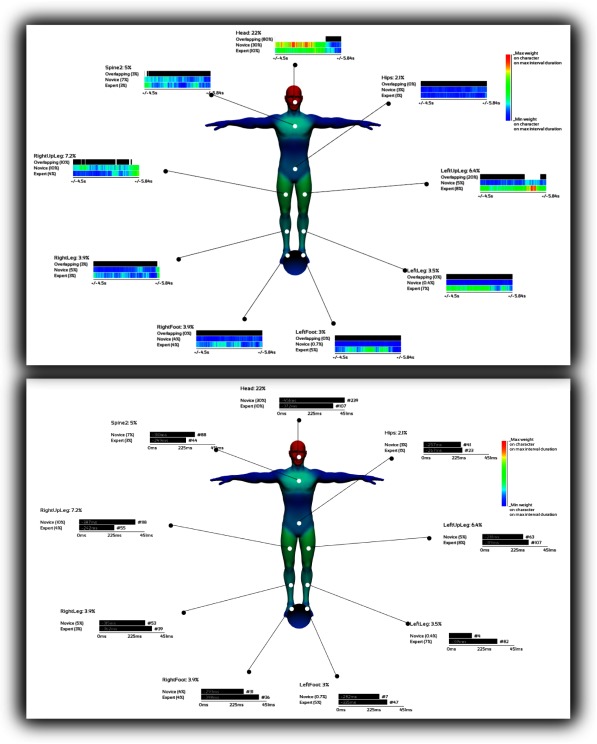
**Penalty anticipation.** Top: Illustration of gaze distribution average for the two groups of viewers (expert goalkeepers (Expert) and field players (Novice)). Bottom: Illustration of viewers' fixations for each group.

**Figure 10.**
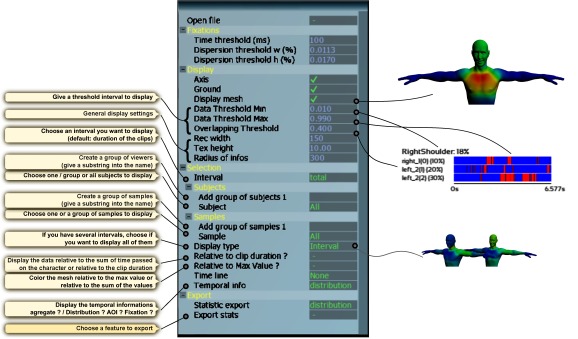
**Graphical User Interface:** Overview of the commands.

**Figure 11.**
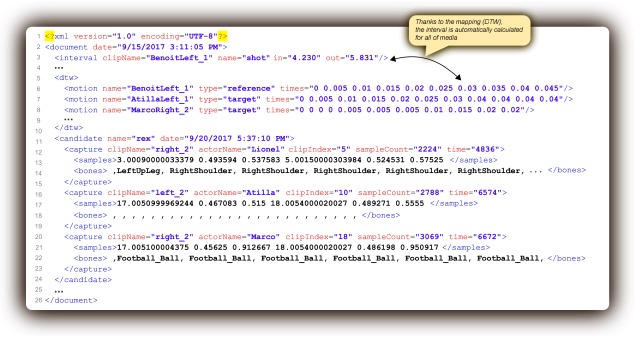
Example of input file.
